# G Protein-Coupled Receptors: Extranuclear Mediators for the Non-Genomic Actions of Steroids

**DOI:** 10.3390/ijms150915412

**Published:** 2014-09-01

**Authors:** Chen Wang, Yi Liu, Ji-Min Cao

**Affiliations:** 1 Department of Medicine, Peking Union Medical College, Chinese Academy of Medical Sciences, Beijing 100730, China; E-Mail: mcherry.f@gmail.com; 2 Department of Endocrinology, Key Laboratory of Endocrinology, Ministry of Health, Peking Union Medical College Hospital, Peking Union Medical College, Chinese Academy of Medical Sciences, Beijing 100730, China; E-Mail: liuyi.dream@gmail.com; 3 Department of Physiology, Institute of Basic Medical Sciences Chinese Academy of Medical Sciences, School of Basic Medicine Peking Union Medical College, Beijing 100005, China

**Keywords:** G protein-coupled receptor, steroid, non-genomic effect

## Abstract

Steroids hormones possess two distinct actions, a delayed genomic effect and a rapid non-genomic effect. Rapid steroid-triggered signaling is mediated by specific receptors localized most often to the plasma membrane. The nature of these receptors is of great interest and accumulated data suggest that G protein-coupled receptors (GPCRs) are appealing candidates. Increasing evidence regarding the interaction between steroids and specific membrane proteins, as well as the involvement of G protein and corresponding downstream signaling, have led to identification of physiologically relevant GPCRs as steroid extranuclear receptors. Examples include G protein-coupled receptor 30 (GPR30) for estrogen, membrane progestin receptor for progesterone, G protein-coupled receptor family C group 6 member A (GPRC6A) and zinc transporter member 9 (ZIP9) for androgen, and trace amine associated receptor 1 (TAAR1) for thyroid hormone. These receptor-mediated biological effects have been extended to reproductive development, cardiovascular function, neuroendocrinology and cancer pathophysiology. However, although great progress have been achieved, there are still important questions that need to be answered, including the identities of GPCRs responsible for the remaining steroids (e.g., glucocorticoid), the structural basis of steroids and GPCRs’ interaction and the integration of extranuclear and nuclear signaling to the final physiological function. Here, we reviewed the several significant developments in this field and highlighted a hypothesis that attempts to explain the general interaction between steroids and GPCRs.

## 1. Introduction

Steroid hormones (hereafter referred to as steroids) modulate many physiological and pathological processes. The classical paradigm of their actions is that intracellular receptors bind to specific steroids and regulate gene expression after they translocate into the nucleus. Such effects take a time lag of hours or even days [1]. However, Selye noted in the early 1950s that some steroids induced biological effects only minutes after their application [2]. In the light of present knowledge, Selye’s observation was actually the first one to describe the non-genomic effect of steroids in detail. Since then, increasing evidence suggests that many important steroid-induced events are rapid and independent of transcription. Studies have effectively launched the field of non-genomic steroids response, nearly extending to all the members of the steroid hormone family. Classic examples of these non-genomic effects include estrogen-mediated blood vessel dilation, progesterone-induced sperm acrosomal action and glucocorticoid-triggered tracheal relaxation [3,4,5,6,7].

The signaling mechanisms responsible for these non-genomic effects are diverse. Although some studies indicate that classical steroid receptors, *i.e.*, nuclear receptors, can initiate second messenger signaling or interact with other cellular signaling components, there is growing body of evidence to suggest that these rapid actions are mediated by specific receptors localized most often to the plasma membrane [8,9]. Membrane impermeable steroid conjugates, such as bovine serum albumin-steroid and polymer-steroid, can still perform these rapid actions, which seem to be the most direct support for the membrane-initiated steroid signaling [10,11,12,13]. However, despite intense research in many laboratories over the past several decades, the precise identities of these receptors in most target tissues are still unknown. Early attempts, via the radio-labeled ligand-receptor purification from isolated membrane fraction, have identified several candidates [14]. Some of them have been cloned further, but none has been unequivocally confirmed as the functional receptor. These studies could be hampered by the lipophilic nature of steroids, resulting in highly nonspecific binding in membrane preparations [15].

To date, several types of candidates have been proposed for the non-genomic effects, including classical steroid receptors localized in the plasma membrane, membrane proteins with steroid-binding domain and G protein-coupled receptors (GPCRs). In terms of classic nuclear receptors, the membrane-bound receptor is thought to be a variant of the cytosolic receptor that results from alternative splicing, promoter switching or post-translational modification [16]. For instance, approximately 5%−10% endogenous estrogen receptor α (ERα) are present at the plasma membrane, which has been suggested to mediate the rapid signaling of estrogen [17]. In respect to the membrane protein with steroid-binding domain, steroids could regulate the inherent function of the membrane protein. An illustrative example is CatSper. This sperm-specific calcium channel that locates in the principle piece of the flagellum has been proven responsible for the progesterone-induced hyperactivation, acrosomal reaction and chemotaxis of the sperm [18,19]. Another example is derived from our recent study, in which we explored the underlying mechanism of how glucocorticoid potentiates the action of catecholamine. In our *ex vivo* tracheal preparation, we showed that glucocorticoid could inhibit the function of extraneuronal monoamine transporter to slow down the elimination of catecholamine [7]. In most cases, however, GPCRs are more appealing candidates to explain the rapid action of steroids. Evidence has been obtained for the involvement of GPCR-G protein signaling in these non-genomic steroids actions. In early studies, pertussis toxin, which locks the inhibitory G (Gi) protein in the guanosine diphosphate (GDP) bound form, could inhibit the non-genomic action of steroids in several models [20,21,22]. Moreover, GPCRs responsible for the action of several steroids, including estrogen, progesterone, androgen and thyroid hormone, were identified and the functional characterizations have proved their physiological relevance to a certain extent. Herein, steroids and corresponding GPCRs that mediate their non-genomic effects are reviewed. A more general hypothesis that tries to explain the interaction between steroids and GPCRs is also discussed.

## 2. Estrogen and G Protein-Coupled Receptor 30 (GPR30)

It is universally appreciated that estrogen receptor (ER)-α and -β mediate most actions of estrogen through gene transactivation and transrepression [23]. However, the physiologically relevant roles of estrogen, via its non-genomic effect, have also been reported in reproductive, renal and vascular systems [4,5]. These actions were not well understood until the GPR30 was identified as a functional receptor of estrogen [24]. GPR30 was discovered by molecular cloning approaches in the late 1990s, although its functional ligand was unknown at that time [25]. In early attempts, demonstrating the specific interaction between estrogen and GPR30 was a difficult challenge, as there is high background binding between the lipophilic ligand and the lipid-rich membrane preparation. With the use of membrane filtration technique, plasma membrane fraction from free ligand could be prepared and the results indicate that estrogen is a highly specific ligand of GPR30, in contrast to other steroids [26]. An important issue of GPR30 study is its subcellular localization. After it was identified as the receptor of estrogen, GPR30 has exhibited many of the expected properties of the long-searched plasma estrogen receptor. However, further studies show that GPR30 is predominately distributed intracellularly, in both cultured cells and tissue specimens [27,28]. Insights regarding the subcellular localization of GPR30 are derived from intracellular trafficking studies. It is now appreciated that there is a constitutive endocytosis of GPR30 [29,30]. These observations indicate that GPR30 is not merely restricted in intracellular membrane, but there is a redistribution process between cell membrane and intracellular membrane. Additionally, the relative abundance of GPR30 on cell membrane is also critical for estrogen signaling in different tissues. Dozens of studies have revealed the downstream signaling of GPR30, which is coupled to stimulatory G (Gs) protein and acts to activate adenylate cyclase and epidermal growth factor receptor [31]. It is intriguing that such signaling pathways are remarkably similar between mammals and fish, which diverged from the vertebrate lineage over 200 million years ago, suggesting that the physiological role of GPR30, as an estrogen receptor, is highly conserved during evolution [32].

Pharmacological and genetic approaches have been used to dissect the physiological and pathophysiological roles of GPR30, mainly in vascular, renal and reproductive systems. The well-documented estrogen-induced vasodilation is thought to be mediated by GPR30 [33]. Furthermore, recent studies further attribute the renoprotective action of estrogen to GPR30 signaling [34]. Moreover, there are dozens of studies concerning the role of GPR30 in estrogen-related carcinogenesis. Activation of GPR30 has been linked to the proliferative behavior of cancer cells from reproductive tissues, including endometrium, ovary, testis and breast [35]. The best evidence supporting an ER-independent role of GPR30 in reproductive cancers comes from studies of its expression in tumor biopsy specimens. GPR30 and ER display distinct patterns of expression and show different association with clinicopathological parameters [28]. Furthermore, the subcellular expression pattern of GPR30 may also reflect different biological features in breast cancer. For instance, nuclear GPR30 expression was shown to be associated with poorly differentiated breast cancer and the triple-negative subtype [36].

## 3. Progesterone and Membrane Progestin Receptor (mPR)

As a gonadal hormone, most non-genomic actions of progesterone are described in germ cells. Progesterone-triggered oocyte maturation is one of the best-characterized, biologically relevant examples of its non-genomic effect [15]. Despite obtaining detailed information on the downstream signaling responsible for oocyte meiosis, the nature of the corresponding membrane receptor for progesterone has been a matter of debate. Early attempts, with the use of molecular cloning approach to screen cDNAs encoding proteins with progesterone-binding domain, identified a *Xenopus* homolog of nuclear PR (xPR), which was thought to mediate the rapid activation of progesterone on oocyte [37,38]. However, functional assays in these early studies were dependent on massive overexpression of xPR, which might cause mislocalization of the receptor to cell membrane. Moreover, no apparent expression of endogenous xPR was detected in the cell membrane. These cast doubts on its physiological significance. With the advance of antibody screening and molecular cloning approaches, the membrane progestin receptor (mPR) was finally identified in 2003 [39,40]. Evidence was further obtained that recombinant mPR could bind with progesterone with the characteristics of a steroid hormone receptor, including high affinity, low capacity and specificity. Phylogenetic analyses indicate that the mPR belongs to a progesterone and adipoQ receptor (PAQR) subfamily, while not classical GPCR [41]. However, after binding with progesterone, mPR could definitively activate G protein and initiate downstream signaling. Physiological concentration of progesterone could lead to a decrease of intracellular cyclic adenosine monophosphate (cAMP) level, which is reversed when treated with pertussis toxin [39]. This observation has been confirmed in several cell models, further supporting the coupling of mPR to Gi protein [42,43].

Although the majority of mPR studies focus on fish and amphibian oocyte maturation, mammalian homologues of mPR have been identified in multiple species, including human, mouse and rat. In addition, a growing number of studies have examined the regulation and activity of mPRs in mammalian physiology [44]. It is clear from these studies that mPR is expressed in various reproductive tissues, from male to female. In males, mPR appears to regulate sperm motility. More concerns have been given to mPR’s role in female reproductive function, including oocyte development, transportation and uterus preparation for implantation. It is noteworthy that upregulated expression of mPR has been observed in breast cancer biopsies and subsequent investigation further confirmed the expression of mPR in biopsies of classic progesterone receptor null breast cancer [45]. These observations may lead to alternative therapies for these endocrine-related malignancies.

The mPR is also highly enriched in the brain and has been demonstrated to be associated with progesterone-induced neuroprotection. This action may be attributed to the neurosteroid, namely allopregnanolone, which is a metabolite of progesterone [46]. Although demonstrated as an allosteric modulator of γ-aminobutyric acid receptor in early reports, allopregnanolone has shown a relatively high affinity for mPR in recent studies [47]. Their interaction could initiate Gi protein mediated downstream signaling. Functional characterizations found that the neuroprotection might be achieved through the inhibitory effect on cell death and apoptosis [48]. Intriguingly, recent studies also illustrated that progesterone, through mPR, could cause a rapid inhibition of gonadotropin releasing hormone (GnRH) secretion [49]. This observation implies that mPR signaling may participate in the regulation of GnRH pulsatility, an important physiological event controlling female fertility. Thus, mPR, highly expressed in brain and genital organs, may cooperatively regulate reproduction.

It is noteworthy that another protein, termed progesterone receptor membrane component 1 (PGRMC1), has also been mentioned as the membrane receptor of progesterone in the literature. PGRMC1 was also cloned in the search for membrane receptor of progesterone [50,51]. The interaction between progesterone and PGRMC1 was mainly demonstrated with the use of microsome membrane containing PGRMC1, or in progesterone-responsive tissue expressing PGRMC1. Biochemical properties and potential roles of this single transmembrane protein (not GPCR) have been reviewed in several papers [52,53]. However, an interesting observation from a recent study shows that PGRMC1 can act as an adaptor protein, assisting the transportation of mPR to the cell surface, and that the progesterone binding and apoptotic functions previously attributed to PGRMC1 are dependent on cell surface expression of mPR [54].

## 4. Androgen and G Protein-Coupled Receptor Family C Group 6 Member A (GPRC6A)

Androgen regulates reproduction and has an important role in anabolic activity in multiple tissues. GPRC6A has been identified as the membrane receptor of androgen and ablation of this Gi protein coupled receptor leads to testicular feminization in male mice [55,56]. These observations may be the first piece of data to inspire further studies concerning the interaction between this receptor and androgen. Detailed studies showed that in established cell model with rapid response to androgen, ablation of GPRC6A, rather than ablation of classic nuclear receptor of androgen, abolished the intracellular signaling responsive to androgen. In addition, GPRC6A-null mice exhibited an impaired response to exogenous administration of androgen, and failed to restore seminal vesicle size, a measure of tissue responsiveness to androgen in this orchectomized GPRC6A-null mouse strain [56]. In terms of the downstream signaling, GPRC6A, coupled with Gi protein, could activate the ERK pathway in both cell culture and tissues [56].

In respect to diseases, GPRC6A was upregulated in various prostate cancer cell lines. GPRC6A activation could stimulate prostate cancer cell proliferation and chemotaxis, while GPRC6A deficiency retarded tumor progression and improved survival in prostate cancer mouse model [57]. These observations raise interesting questions about the interrelationship between GPRC6A and classic androgen receptor in prostate cancer responsive to androgen.

In contrast to the relationship between estrogen and GPR30 and between progesterone and mPR, the interaction between androgen and GPRC6A is not specific. GPRC6A is also activated by extracellular calcium, amino acids, and osteocalcin [58]. These sensing properties of GPRC6A make it as a coordinator for nutritional and hormonal anabolic signals, rather than a sole effecter for androgen. This may be physiologically relevant, as the picture of androgen-mediated non-genomic effects seems to be more complex than other steroids. The mechanism which produces the effect varies in different cell types. A possible explanation is that the non-genomic actions of androgen are likely linked with other signaling, such as calcium signaling. They can function independently, or in tandem, resulting in the final biological effects [59].

However, researchers may prefer the classic idea that a unique membrane receptor exists, mediating the non-genomic actions of androgen. Encouragingly, Thomas and colleagues, using a strategy similar to mPR identification, found a novel 7-transmembrane protein with characteristics of membrane androgen receptor (mAR) [60,61]. This protein is highly specific for androgen binding, with low or no affinity for other steroids. Interestingly, it shows high sequence similarity with zinc transporter ZIP9 subfamily, rather than the classic GPCR. However, androgen does activate a Gs protein coupled to ZIP9, consistent with an increase in cAMP level. The model they used to test the non-genomic action of androgen was serum starvation-induced cell death and apoptosis, firstly in croaker ovarian follicle cells, and then extended to breast cancer and prostate cancer cell lines. Their studies showed that androgen could promote serum starvation-induced cell apoptosis in these cell lines. A unique property of ZIP9 identified in their studies is that androgen can elicit both Gs protein-mediated signaling and elevation of intracellular zinc concentration. These two responses may both contribute to the final effect. Nevertheless, the physiological significance of androgen-triggered apoptosis is unclear. Whether other non-genomic actions of androgen are also related to this protein requires further studies. Furthermore, controversial effects of androgen on reproductive malignancy, through different receptors, need also to be clarified.

## 5. Thyroid Hormone and Trace Amine Associated Receptor 1 (TAAR1)

Although thyroid hormones, including thyroxine (T_4_) and triiodothyronine (T_3_), are structurally different from steroids, they are always included when discussing actions of steroids, as their classic receptors (thyroid receptor, TR) belong to nuclear receptor superfamily, and thyroid hormones can also elicit non-genomic effects [3,5].

Similar to classic steroids, thyroid hormones bind with TR to modulate transcription of target genes, the effect of which manifests over hours to days. Meanwhile, rapid, non-genomic effects of thyroid hormones are also well documented. Such effects concern calcium influx, oxygen consumption and cardiac functions [62]. A possible mediator of these effects was identified in 2004, and was confirmed as a GPCR. This GPCR, named trace amine-associated receptor 1 (TAAR1), binds to 3-iodothyronamine, a thyroid hormone metabolite, rather than classic thyroid hormones [63]. The downstream events involved in TAAR signaling are not fully understood. In heterologous cellular systems, TAAR1 could couple with Gs protein, resulting in adenylate cyclase activation, although other G proteins, including Gq and Gα16, might also be utilized by TAAR1 [64].

It should be emphasized that 3-iodothyronamine is a naturally occurring derivative of thyroid hormone, which has been identified in tissue homogenates with the use of liquid chromatography and tandem mass spectrometry. In addition, TAAR1 is shown to have a very rich pharmacology which can be activated by compounds of different classes, including dopaminergic, adrenergic and serotoninergic [65]. Such a promiscuous interaction between ligand and receptor may be due to the less rigorous ligand-binding pocket [66]. In terms of 3-iodothyronamine, TAAR1 recognizes this amine via hydrogen binding to its amine group [67], which is very different from the recognition of steroids by their known receptors [68].

Further studies have been performed to evaluate the functional consequence of 3-iodothyronamine exposure to different organs. In mice, intraperitoneal injection of 3-iodothyronamine induced a rapid decrease of body temperature [63]. In isolated heart, infusion of 3-iodothyronamine resulted in fast inhibition of heart contractility and rate [63]. In thyroid cells, a slight inhibition of glucose uptake was observed in the presence of 3-iodothyronamine [69]. These data suggest that 3-iodothyronamine could employ TAAR1, a GPCR, to achieve rapid actions. However, these effects are opposite to those produced on a longer time scale by traditional thyroid hormones, and thus may represent an unrecognized branch of thyroid hormone signaling. Further investigations are still required to clarify the physiological significance of 3-iodothyroamine-TAAR1 signaling [70].

## 6. A General Paradigm of Interactions between G Protein-Coupled Receptors and Steroids

Although GPCR signaling has been highly implied in the non-genomic effects of various steroids, only limited cases have been fully delineated, with the identification of corresponding receptors, as mentioned above (Table 1). Thus, there may be a more general interaction between steroids and GPCRs, rather than the classic single ligand-single receptor paradigm. A question is how these highly hydrophobic steroids interact with GPCRs that usually do not contain known steroid-binding domain. The answer may be derived from a consensus binding motif that is originally defined for cholesterol, which shares the mother nucleus with steroids. This motif, named cholesterol recognition/interaction amino acid consensus sequence (CRAC), is thought to be conserved in about one-third of class A GPCRs [71,72]. Moreover, cholesterol and a variety of sterol analogues, via interaction with CRAC, have been shown to impact the agonist-binding affinity of GPCR [73]. In light of these data, we recently proposed a hypothesis that the non-genomic effect of steroids is mediated by the interaction between steroids and GPCRs via CRAC, which further influences the receptor activity [74]. The interaction between steroids and CRAC is specific, while the involved GPCRs need not be specific, *i.e.*, dozens of GPCRs bearing CRAC could be regulated by the steroids simultaneously. This pan-interaction could accommodate the unexplained controversies in literatures: (i) different steroids can exert a similar non-genomic effect, as both progestin and androgen can promote oocyte maturation [14,15]; (ii) for a single steroid, various downstream signaling pathways have been implicated, including adenylate cyclase-protein kinase A (PKA) or phospholipase C (PLC)-protein kinase C (PKC) [5]; (iii) different effects are initiated by same steroid in various models, which can be explained by the compositional difference of GPCRs’ pool expressed in distinct cell types; (iv) there is no strict enantioselectivity for the steroid effect in accordance with the observed promiscuity of cholesterol-binding site for a series of sterol analogues [75]. This hypothesis can be regarded as a general mechanism to explain the highly variable non-genomic effects and also the multiple failures in the search for specific membrane receptors for most steroids.

**Table 1:** Steroids, non-genomic effects and corresponding G protein-coupled receptors (GPCRs).

Steroids	Non-Genomic Effects	GPCRs
Estrogen	Cardio-renal physiology: vasodilation [33], renoprotection [34] Reproductive physiology: mammary gland development, oocyte maturation, endometrial cell growth and myometrial contraction [35] Reproductive cancer: breast, ovary, testis, endometrial and uterine cancer cell proliferation and survival [35,36]	GPR30
Progesterone	Reproductive physiology: fish and amphibian oocyte maturation; teleost, mouse and human sperm motility [39,43] Reproductive cancer: breast, ovarian, cervical cancer cell proliferation, survival and invasion [44,45] Neural physiology: neuroprotection [48], hypothalamus hormone (e.g., GnRH) release [49]	mPR
Androgen	Reproductive physiology: hypothalamus-pituitary-gonadal gland axis, e.g., luteinizing hormone release; seminal vesicle development [55,56] Reproductive cancer: prostate cancer cell proliferation, survival and invasion [57]	GPRC6A
	Reproductive physiology: croaker ovarian follicle cell apoptosis [60] Reproductive cancer: breast and prostate cancer cell apoptosis [61]	ZIP9
3-Iodothyronamine	Cardiac physiology: negative inotropic and chronotropic action [63] Metabolism: body temperature decrease [63], glucose uptake inhibition in thyroid cells [69]	TAAR1

In terms of several specific GPCRs aforementioned, whether there are functional CRACs still requires further molecular modeling and even structural biological studies. In principle of our hypothesis, we prefer the idea that a GPCR restricted for a certain steroid hormone may have a unique ligand-binding pocket, as in the cases of GPR30 and mPR. In contrast, a GPCR that is responsible for various ligands may bear a promiscuous docking site for the ligand, *i.e.*, CRAC.

## 7. Conclusions

The non-genomic effect of steroids is membrane receptor-mediated signaling, which is distinct from the classical action of steroids via their nuclear receptors. Increasing evidence suggests that the nature of these membrane receptors is consistent with the properties of GPCRs. Indeed, several physiologically relevant GPCRs, including GPR30 for estrogen, mPR for progesterone, GPRC6A and ZIP9 for androgen, and TAAR1 for thyroid hormone, have been identified and demonstrated to be responsible for the non-genomic effects of specific steroids. However, a more general paradigm is still required to explain the highly variable interaction between steroids and GPCRs. Therefore, a hypothesis has been proposed that the interaction between steroids and CRAC of unconstrained GPCRs may contribute to the non-genomic effects to some extent. Further studies are needed to validate this hypothesis. Moreover, both the confirmed membrane receptors of steroids and the CRAC in various GPCRs are potential targets for compound development, which could be useful not only for further functional studies, but also for therapeutic interventions.
